# In vitro study of color stability of polycrystalline and monocrystalline ceramic brackets

**DOI:** 10.1590/2176-9451.19.4.114-121.oar

**Published:** 2014

**Authors:** Cibele Braga de Oliveira, Luiz Guilherme Martins Maia, Ary Santos-Pinto, Luiz Gonzaga Gandini Júnior

**Affiliations:** 1 MSc in Orthodontics, State University of São Paulo (UNESP).; 2 Professor, Department of Orthodontics, UNIT/SE.; 3 Full professor, Department of Orthodontics, State University of São Paulo (UNESP).

**Keywords:** Color, Ceramics, Orthodontic brackets

## Abstract

**Objective:**

The aim of this *in vitro* study was to analyze color stability of monocrystalline and polycrystalline ceramic brackets after immersion in dye solutions.

**Methods:**

Seven ceramic brackets of four commercial brands were tested: Two monocrystalline and two polycrystalline. The brackets were immersed in four dye solutions (coffee, red wine, Coke and black tea) and in artificial saliva for the following times: 24 hours, 7, 14 and 21 days, respectively. Color changes were measured by a spectrophotometer. Data were assessed by Multivariate Profile Analysis, Analysis of Variance (ANOVA) and Multiple Comparison Tests of means.

**Results:**

There was a perceptible change of color in all ceramic brackets immersed in coffee (ΔE* Allure = 7.61, Inspire Ice = 6.09, Radiance = 6.69, Transcend = 7.44), black tea (ΔE* Allure = 6.24, Inspire Ice = 5.21, Radiance = 6.51, Transcend = 6.14) and red wine (ΔE* Allure = 6.49, Inspire Ice = 4.76, Radiance = 5.19, Transcend = 5.64), but no change was noticed in Coke and artificial saliva (ΔE < 3.7).

**Conclusion:**

Ceramic brackets undergo color change when exposed to solutions of coffee, black tea and red wine. However, the same crystalline structure, either monocrystalline or polycrystalline, do not follow the same or a similar pattern in color change, varying according to the bracket fabrication, which shows a lack of standardization in the manufacturing process. Coffee dye produced the most marked color changes after 21 days of immersion for most ceramic brackets evaluated.

## Abstract (pt)

**Objetivo:**

esse estudo objetivou analisar, *in vitro*, a estabilidade de cor de braquetes cerâmicos monocristalinos e policristalinos após imersão em soluções corantes.

**Métodos:**

sete braquetes cerâmicos de incisivo central superior direito, de quatro marcas comerciais, foram testados: dois monocristalinos e dois policristalinos. Os braquetes foram imersos em quatro soluções corantes (café, vinho tinto, Coca-Cola e chá preto) e em saliva artificial, separadamente, nos seguintes tempos: 24 horas, 7, 14 e 21 dias. As alterações de cor foram mensuradas por espectrofotômetro de refletância. Os dados foram avaliados pela Análise de Perfis Multivariados, Análise de Variância e teste de comparação múltipla de médias.

**Resultados:**

houve alteração perceptível de cor em todos os braquetes cerâmicos após 21 dias de imersão nas soluções de café (ΔE* Allure = 7,61; Inspire Ice = 6,09; Radiance = 6,69; Transcend = 7,44), chá preto (ΔE* Allure = 6,24; Inspire Ice = 5,21; Radiance = 6,51; Transcend = 6,14) e vinho tinto (ΔE* Allure = 6,49; Inspire Ice = 4,76; Radiance = 5,19; Transcend = 5,64), porém, a alteração não foi perceptível para a Coca-Cola e saliva artificial (ΔE* < 3,7).

**Conclusão:**

braquetes cerâmicos sofrem alteração de cor quando em contato com café, chá preto e vinho tinto. No entanto, os braquetes de mesma constituição cristalina, sejam monocristalinos ou policristalinos, não seguem um padrão semelhante de alteração de cor, mas variam de acordo com o fabricante, o que mostra uma falta de padronização no processo de produção desses braquetes. O café foi a solução corante que mais produziu alteração de cor após 21 dias de imersão, para a maioria dos braquetes cerâmicos avaliados.

## INTRODUCTION

The first esthetic brackets appeared in the 70's and were made from polycarbonate, a plastic material. Although these brackets were reasonably esthetic, this material did not present suitable properties for clinical use. Several studies showed clinical problems such as deformation and structural weakness, poor adhesion and poor stain resistance during treatment.^1,2,3^

In the mid-1980s, other types of material were tested to meet the esthetic needs of the orthodontic market, and that was when esthetic ceramic brackets appeared. These brackets are mainly made from aluminum oxide and are available in two forms according to the manufacturing process: polycrystalline or monocrystalline.^4,5^

Polycrystalline or alumina polycrystalline brackets are made of aluminum oxide crystals fused at high temperatures (near 1950°C).^6^ Monocrystalline brackets are made of a single crystal produced from the combination of particles of aluminum oxide fused at a higher temperature (2100°C) and cooled slowly, thus enabling thorough control of crystallization.^4,7^

Thus, the manufacturing process produces translucent and nontranslucent ceramic brackets. Monocrystalline brackets are included in the translucent brackets group while polycrystalline brackets are nontranslucent.^8^ The translucency of monocrystalline brackets is due to the structure of a single crystal that provides passage of light. Polycrystalline brackets are not translucent because their structure presents lack of boundaries between the crystals and impurities incorporated during the manufacturing process, thereby hindering passage of light.^9^

To have a good esthetic appearance, nontranslucent brackets need to be similar in color and fluorescence to the underlying tooth, whereas translucent brackets need to have sufficient translucency so as to allow the color and fluorescence of the tooth to pass through them. However, it is essential that both have good color stability.^8^

Even if esthetics is the only advantage of ceramic brackets in relation to metal brackets, they are not color stable in the long term. As reported by some authors, the color of these accessories changes in the oral environment due to staining from substances containing pigments commonly found in food and drinks.^3,6,10,11,12^

Nevertheless, only a few studies have been conducted to investigate the color changes and the factors that lead to such changes. Therefore, this study aims to analyze *in vitro* the color stability of monocrystalline and polycrystalline esthetic ceramic brackets after immersion in dye solutions commonly present in food and drinks in order to know if the crystal structure of these brackets follow a similar pattern of color change.

## MATERIAL AND METHODS

The sample comprised maxillary right central incisors ceramic brackets, slot size 0.022 x 0.028-in in Roth prescription. Four commercial brands were selected: Two brands were monocrystalline brackets - Inspire Ice from Ormco^®^ (Orange, California) and Radiance from American Orthodontics^®^ (Sheboygan, Wisconsin); and two were polycrystalline brackets - Allure MB from GAC^®^ (Bohemia, New York) and Transcend from 3M Unitek^®^ (Monrovia, California). To prevent the glue surfaces of different brands from interfering in the staining process, all surfaces were worn with a diamond drill bit. Seven brackets of each brand were tested.

The brackets were immersed in solutions of coffee, dry red wine, Coke, black tea and artificial saliva (control group) (Table 1). Each one of these solutions was distributed into glass chambers with partitions to separate the different brands of brackets. These containers were placed in an incubator at a temperature of 37°C wrapped in black plastic bags to eliminate the interference of light. The solutions were changed every 24 hours and their pH was measured with a pH meter (Model 8010, Qualxtron) at each change to check whether it remained the same.

**Table 1:** Solutions, brands, pH values and preparation methods.

Solution	pH	Brand	Preparation
Coffee	5	Nescafé (Nestlé, Brazil Ltda, Brazil)	Solution prepared with 50 g of instant coffee added to 200 ml of boiling distilled water.
Red wine	3.3	Dry red wine (Canção - Serra Gaúcha, Rio Grande do Sul, Brazil)	Solution ready for consumption
Black tea	5.2	Black tea sachet (Leão Junior S.A., Brazil)	Solution prepared with one black tea sachet immersed in 200 ml of boiling distilled water
Coke	2.4	Coke (Coca-Cola Co.)	Solution ready for consumption
Artificial saliva	7	Artificial saliva (Farmácia Santa Paula – Araraquara, São Paulo, Brazil).	Prepared solution (neutral pH, tasteless and odorless )

* All solutions were distributed into containers at ambient temperature.

The color parameters of brackets were measured at the following times: T_0_ (initial measurement), T_1_, T_2_, T_3_ and T_4_ (brackets immersed in dye solution for 24 hours, 7, 14 and 21 days, respectively). Before each color reading, brackets were washed with distilled water and blotted dried to remove any residual waste from the dyes on the brackets.

Color measurements of each group of brackets were obtained using a portable reflectance spectrophotometer Spectro-guide (Byk Gardner^®^, Columbia, USA). Spectro-guide measures the intensity of each wavelength of light reflected by the sample when illuminated by a polychromatic light (illuminant D65) emitted from the device at an angle of 45°. Light reflected from the sample is captured by a viewing angle of 0°. The measuring aperture diameter size was 3 mm. Tristimulus values ​​(L*, a* and b*) were supplied by the device from the captured light.

Potential color changes of brackets were measured in accordance with the Commission Internationale de l'Eclairage (CIE) L*, a*, b* (LAB) color scale.^13^ This color measurement system^14^ quantitatively determines color by using three parameters (L*, a* and b*). On the CIELAB color scale, L* is a measure of brightness of an object. It is quantified on a scale in which black has a L* value of zero; and light, which is totally reflected, has a L* value of 100. On the same scale, a* accounts for the amount of red (+ a*) and green (-a*), whereas b* accounts for the amount of yellow (+ b*) and blue (- b*). Color change (ΔE*) was calculated using the equation: ΔE* = [(ΔL *)^2^ + (Δa *)^2^ + (Δb *)^2^] 1/2. Changes in color parameters ΔL*, Δa* and Δb* were obtained by subtracting the final values ​​from the baseline (T_0_).

For color measurements, brackets were positioned in a matrix of white silicone rubber from Redelease^®^ (4 matrices were made for the four brands of brackets) coupled to a positioner where the spectrophotometer was embedded. Thus, the brackets and the spectrophotometer were always in the same position for all measurements.

### Statistical analysis

To evaluate the error of the method, two measurements were made for each variable. Reproducibility was assessed by means of the Intraclass Correlation Coefficient (ICC).

Multivariate Profile Analysis, carried out by means of the Pillai Trace test, was used to evaluate the effect of time.

Analysis of Variance (ANOVA) with one classification criterion and Bonferroni Test of Multiple Comparison of means were used to define any statistical difference of color change between brands and between solutions. These analyses were preceded by a test of homogeneity of variances. Should homogeneity of variances be rejected, ANOVA was replaced by Brown-Forshyte test and the Multiple Comparison Tests of means was conducted with the Tamhane test.

The statistical software SPSS version 16.0 (Statistical Package for Social Sciences, SPSS Inc., Chicago, IL, USA) was used to tabulate and analyze data. Statistical significance was adopted at 95% confidence interval.

## RESULTS

Results of intraclass correlation coefficients (ICC) revealed that the method for measuring the color of ceramic brackets was effective. A high degree of reproducibility as obtained for all parameters of color (L*, a*, b*), thus indicating a negligible method error (range limit top and bottom of the ICC: 0.8 to 1.00).

Table 2 presents the results of the multivariate tests for assessing color change over time. Results show that color of ceramic brackets changes over time. Furthermore, there is no similarity of color change over time among all brackets. A significant difference in bracket staining between solutions was also observed over immersion time. Thus, color change over time depends on a brand-solution combination.

**Table 2:** Pillai trace multivariate test for significance of color change. Intra individual factor = time.

Effect	Pillai trace	F test				Power
		F	df1	df2	p	
Time	0.945	1419.643	3	250	<0.001	> 0.999
Time * brand	0.776	29.296	9	756	<0.001	> 0.999
Time * solution	1.441	58.208	12	756	<0.001	> 0.999
Time * brand * solution	0.56	4.816	36	756	<0.001	> 0.999

Note: df1 = numerator degrees of freedom; df2 = denominator degrees of freedom.

### Comparative study of ceramic brackets staining by solution and immersion time

**Figure 1:**
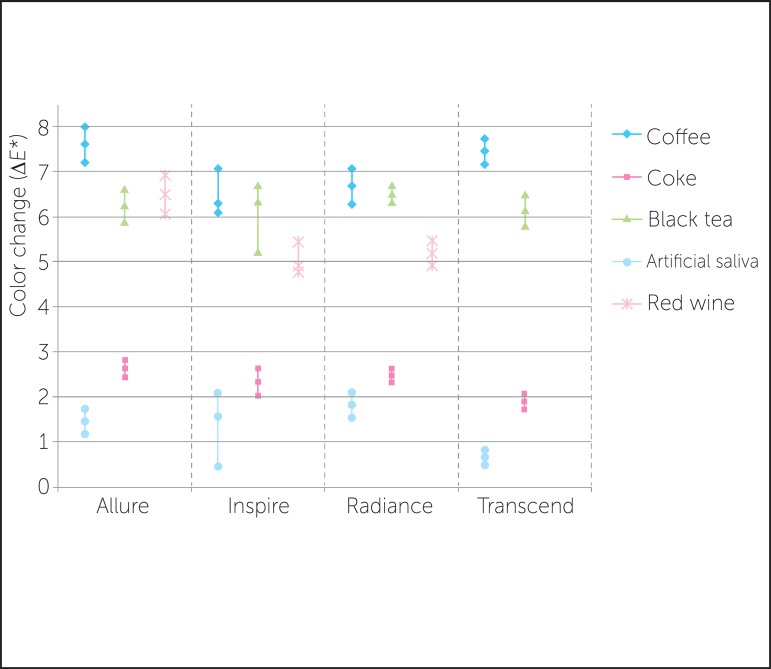
Mean values and 95% confidence intervals of color change by brand in coffee, black tea, red wine, Coke and artificial saliva solutions.

Bracket brands (two monocrystalline and two polycrystalline) were assessed for color change produced by each solution within each time period. Results are shown in Table 3 and Figure 1.

**Table 3:** Mean and standard deviation of color change (DE*) dof ceramic brackets in each solution and time, result of the Variance Analysis and multiple comparison of means.

Time of immersion	ALLURE	INSPIRE ICE	RADIANCE	TRANSCEND	ANOVA	
	ΔE* Mean ± SD	ΔE* Mean ± SD	ΔE* Mean ± SD	ΔE* Mean ± SD	F	P
**ARTIFICIAL SALIVA**						
24 hours	1.26^A^ ± 0.20	1.07^AB^ ± 0.13	0.81^B^ ± 0.47	0.29^C^ ± 0.14	16.79	0.000
7 days	1.27A^B^ ± 0.27	1.10^A^ ± 0.32	1.68^B^ ± 0.48	0.41^C^ ± 0.09	19.35	0.000
14 days	1.43^A^ ± 0.29	1.06^A^ ± 0.12	1.37^A^ ± 0.40	0.44^B^ ± 0.17	20.09	0.000
21 days	1.45^A^ ± 0.30	0.46^B^ ± 0.10	1.82^C^ ± 0.30	0.64^B^ ± 0.17	53.58	0.000
**COKE**						
24 hours*	1.40^A^ ± 0.21	2.22^B^ ± 0.10	1.29^A^ ± 0.14	1.65^AB^ ± 0.78	7.01	0.014
7 days	2.43^A^ ± 0.20	2.02^B^ ± 0.21	2.29^AB^ ± 0.13	1.53^C^ ± 0.30	23.03	0.000
14 days	2.50^A^ ± 0.33	2.13^BC^ ± 0.19	2.43^AC^ ± 0.18	1.89^B^ ± 0.13	11.11	0.000
21 days	2.63^A^ ± 0.21	2.02^B^ ± 0.25	2.48^A^ ± 0.18	1.89^B^ ± 0.18	20.61	0.000
**COFFEE**						
24 hours	2.28 ± 0.27	2.44 ± 0.18	2.16 ± 0.34	2.47 ± 0.24	2.14	0.126
7 days	5.16^A^ ± 0.48	3.57^B^ ± 0.34	5.33^A^ ± 0.30	5.05^A^ ± 0.23	37.75	0.000
14 days	5.47^A^ ± 0.32	4.97^B^ ± 0.37	5.91^A^ ± 0.34	5.94^A^ ± 0.22	14.44	0.000
21 days	7.61^A^ ± 0.44	6.09^B^ ± 0.38	6.69^C^ ± 0.42	7.44^A^ ± 0.31	22.59	0.000
**BLACK TEA**						
24 hours	0.99^A^ ± 0.24	1.74^B^ ± 0.22	1.27^A^ ± 0.25	1.19^A^ ± 0.15	14.84	0.000
7 days	3.23^A^ ± 0.34	2.17^B^ ± 0.20	3.51^A^ ± 0.24	3.15^A^ ± 0.13	41.09	0.000
14 days	4.77^A^ ± 0.23	4.29^B^ ± 0.28	5.44^C^ ± 0.24	4.73^A^ ± 0.30	22.72	0.000
21 days	6.24^A^ ± 0.39	5.21^B^ ± 0.38	6.51^A^ ± 0.20	6.14^A^ ± 0.39	18.42	0.000
**RED WINE**						
24 hours*	3.07^A^ ± 0.51	3.62^A^ ± 0.27	1.83^B^ ± 0.27	2.04^B^ ± 0.14	47.30	0.000
7 days	5.21^A^ ± 0.44	3.63^B^ ± 0.39	4.24^C^ ± 0.43	3.50^B^ ± 0.24	28.84	0.000
14 days	5.76^A^ ± 0.33	4.53^B^ ± 0.57	5.26^AC^ ± 0.21	4.91^BC^ ± 0.29	13.67	0.000
21 days	6.49^A^ ± 0.47	4.76^B^ ± 0.69	5.19^BC^ ± 0.31	5.64^C^ ± 0.33	16.94	0.000

Notes: 1) In times marked with a *, brands are not equal. In this case, ANOVA was replaced by Brown-Forsythe test. 2) Same letters account for statistically similar means. Letters are not displayed when the result of ANOVA was not significant or when multiple comparison of means was not able to detect different means.

There was an overall pattern of increasing color change for all brands. However, brackets with the same crystal formation do not follow the same or similar patterns in terms of color change. In other words, monocrystalline or polycrystalline structures do not interfere in how brackets are stained. Furthermore, Inspire Ice bracket had the lowest color change mean after 21 days.

### Comparative study of dye solutions for color change produced on brackets within each immersion time.

In this analysis, solutions were compared for color changes produced on brackets of each brand tested within each time period, as shown in Table 4.

**Table 4:** Mean and standard deviation of color change (DE*) produced by solutions on ceramic brackets over time; result of Variance Analysis and multiple comparison of means.

Time of immersion	ARTIFICIAL SALIVA	COKE	COFFEE	BLACK TEA	RED WINE	ANOVA	
	ΔE * Mean ± SD	ΔE * Mean ± SD	ΔE * Mean ± SD	ΔE * Mean ± SD	ΔE * Mean ±SD	F	P
**ALLURE**							
24 hours	1.26^A^ ± 0.2	1.4^A^ ± 0.21	2.28^B^ ± 0.27	0.99^A^ ± 0.24	3.07^B^ ± 0.51	53.53	0.000
7 days	1.27^A^ ± 0.27	2.43^B^ ± 0.2	5.16^C^ ± 0.48	3.23^D^ ± 0.34	5.21^C^ ± 0.44	158.86	0.000
14 days*	1.43^A^ ± 0.29	2.5^B^ ± 0.33	5.47^C^ ± 0.32	4.77^D^ ± 0.23	5.76^C^ ± 0.33	280.48	0.000
21 days	1.45^A^ ± 0.3	2.63^B^ ± 0.21	7.61^C^ ± 0.44	6.24^D^ ± 0.39	6.49^D^ ± 0.47	359.29	0.000
**INSPIRE ICE**							
24 hours	1.07^A^ ± 0.13	2.22^B^ ± 0.1	2.44^B^ ± 0.18	1.74^C^ ± 0.22	3.62^D^ ± 0.27	173.66	0.000
7 days*	1.1^A^ ± 0.32	2.02^B^ ± 0.21	3.57^C^ ± 0.34	2.17^B^ ± 0.2	3.63^C^ ± 0.39	90.93	0.000
14 days*	1.06^A^ ± 0.12	2.13^B^ ± 0.19	4.97^C^ ± 0.37	4.29^D^ ± 0.28	4.53^CD^ ± 0.57	173.26	0.000
21 days	0.46 ^A^ ± 0.1	2.02B ± 0.25	6.09^C^ ± 0.38	5.21^D^ ± 0.38	4.76^D^ ± 0.69	233.33	0.000
**RADIANCE**							
24 hours	0.81^A^ ± 0.47	1.29^BCD^ ± 0.14	2.16^B^ ± 0.34	1.27^C^ ± 0.25	1.83^D^ ± 0.27	33.9	0.000
7 days	1.68^A^ ± 0.48	2.29^B^ ± 0.13	5.33^C^ ± 0.3	3.51^D^ ± 0.24	4.24^E^ ± 0.43	497.23	0.000
14 days	1.37^A^ ± 0.4	2.43^B^ ± 0.18	5.91^C^ ± 0.34	5.44^D^ ± 0.24	5.26^D^ ± 0.21	706.24	0.000
21 days*	1.82^A^ ± 0.3	2.48^B^ ± 0.18	6.69^C^ ± 0.42	6.51^D^ ± 0.2	5.19^E^ ± 0.31	713.58	0.000
**TRANSCEND**							
24 hours	0.29^A^ ± 0.14	1.65^A^ ± 0.78	2.47^B^ ± 0.24	1.19^A^ ± 0.15	2.04^B^ ± 0.14	19.64	0.000
7 days*	0.41^A^ ± 0.09	1.53^B^ ± 0.3	5.05^C^ ± 0.23	3.15^D^ ± 0.13	3.5^E^ ± 0.24	131.54	0.000
14 days	0.44^A^ ± 0.17	1.89^B^ ± 0.13	5.94^C^ ± 0.22	4.73^D^ ± 0.3	4.91^D^ ± 0.29	358.04	0.000
21 days	0.64^A^ ± 0.17	1.89^B^ ± 0.18	7.44^C^ ± 0.31	6.14^C^ ± 0.39	5.64^D^ ± 0.33	415.68	0.000

Note: 1) In times marked with a *, brands are not equal. In this case ANOVA was replaced by Brown-Forsythe test. 2) Same letters account for statistically similar means. Letters are not displayed when the result of ANOVA was not significant or when multiple comparison of means was not able to detect different means.

Color change was considered clinically significant only for values ​​of ΔE* > 3.7.^15,16,17^ Thus, artificial saliva and Coke solutions did not produce major changes distinguished by the naked eye. Conversely, coffee, black tea and red wine promoted visible changes, in general, from the 14^th^ day of immersion on.

Coffee dye solution produced the most marked color changes after 21 days of immersion for most brackets, except for Transcend brackets that, despite having the highest average, were statistically similar to black tea.

## DISCUSSION

The results of this study led to the conclusion that all ceramic brackets change in color. However, brackets with the same crystal formation did not follow the same or similar patterns of color change when exposed to the same dye solutions under the same conditions. Thus, the degree of staining was different in several brands, and monocrystalline or polycrystalline structure did not affect staining, thereby showing that esthetic behavior depends on the bracket manufacturer.

In the literature, there are few studies about color changes of different esthetic ceramic brackets after immersion in dye solutions. According to them,^16-19^ the color of ceramic brackets changes over time when exposed to potentially dye solutions commonly present in people's diet. In addition, staining is cumulative, it increases as the time of exposure to the coloring elements increases. Nevertheless, only a few studies were comparable with the results of this study, given that most of them compare ceramic and plastic brackets.

Among them, a recent work^19^ obtained similar findings to our study. The authors confirmed that the crystal structure of ceramic brackets has no effect on staining. This conclusion was obtained after confirming that Inspire Ice and Radiance, two monocrystalline brackets, had the lowest and highest values of color change, respectively.

Other authors^20^ are in agreement with these findings. They report that brackets with the same composition made by different manufacturers had different behaviors in color change. This shows a discrepancy in the manufacturing process of brackets and its influence on their esthetic performance.

Only one research^16^ yielded opposite results. According to the authors, color stability of monocrystalline and polycrystalline ceramic brackets remained statistically equal after 14 days. Nevertheless, color was measured at the base brackets worn by drill, thereby not corresponding to the actual surface area exposed to the oral environment or to the entire surface conditions produced by the manufacturer.

According to Yu and Lee,^20^ variation in staining between brackets with the same crystalline formation can be explained by lack of evidence proving that brackets classified within the same composition category, but made by different manufacturers, are actually made of the same material. Moreover, they also raised the possibility that size, shape and roughness could be the cause of this divergence in the optical properties of ceramic brackets. Lee^15^ corroborates the aforementioned work by asserting that bracket surface, size and type of accessories may influence color stability . He also suggests that further studies on the influence of surface on color stability be conducted in order to yield results that can be used to develop esthetic brackets with improved color stability. However, there is no scientific evidence supporting these theories.

For the present study, Inspire Ice bracket had the lowest mean value of color change in most solutions. However, there was a variation in staining, depending on the interaction between the bracket brand and solution. For this reason, we cannot determine which bracket has the best or worst color stability. These results confirm previous studies^16,19,20^ that assessed staining of ceramic brackets and also found many levels of staining, depending on the solution assessed.

The clinical relevance of color changes in the ceramic brackets should also be addressed in terms of dye potential. For Cosmetic Dentistry, color change greater than 2 is already visible for all observers.^21^ Nevertheless, we adopted a parameter of 3.7. This value is based on the limits of color change that are clinically visible and used in studies with ceramic facets ^22^ and esthetic brackets.^15,16^

Considering the value ​​of ΔE* > 3.7 as clinically significant staining, artificial saliva and Coke did not produce color changes that were perceptible to the naked eye. Conversely, coffee, black tea and red wine produced visible changes (ΔE* > 3.7) from the 14^th^ day of immersion on. Coffee solution produced the most marked color changes for all brackets after 21 days of immersion, except for Transcend brackets that, despite having the highest mean value, were statistically similar to black tea.

Our results are in agreement with a previous study^17^ that showed that mean values above 3.7 were obtained for Transcend and INVU ceramic brackets immersed in coffee solution after 14 days. Artificial saliva did not visibly alter the color of these brackets.

Different values ​​of perceptible color changes were used in a different study^18^ which assert that the range of 5 < ΔE* < 10 is noticeable. The authors also claim that mean values greater than 10 account for indisputable discoloration of brackets. Thus, they concluded that all brackets immersed in black tea, coffee or red wine showed similar reactions with a marked increase in discoloration after 5 days. This study corroborates our results; however, with a staining power achieved within a shorter period of time, since, after 24 hours, color change was observed with ΔE* > 3.7 for all brackets.

Divergent results were found in another study^16^ in which color change values were below clinically significant values (ΔE* > 3.7) for all brackets in all solutions. However, these values ​​cannot be compared to other studies, since their spectrophotometric evaluation was performed on the base of worn brackets, unlike most studies that measured color on the vestibular surface of these accessories.

It has been shown that even though Coke has a lower pH value that can damage surface integrity of material, it does not promote clinically significant color changes like coffee and black tea do, possibly due to lack of yellow dye in its constitution.^23^ Coffee and tea have yellow dye, but with different polarities, which differs in their interaction with material surface. Park et al^24^ also showed that pH was not the main element responsible for color changes. According to the authors, the amount and type of pigment was the main reason, thereby confirming the previous study. Studies on color change and ceramic brackets do not usually investigate the physical and chemical interaction between dye solutions and material components. Fort his reason, additional studies are warranted to further investigate this topic.

By comparing the *in vitro* results of this study with the clinical practice, some limitations are encountered, namely: Complex flora of the oral cavity and its byproducts, as well as the buildup of biofilm on tested material.^25^ Therefore, the present study as well as other *in vitro* studies showed overestimated values ​​of color change. Since no *in vivo* studies have been conducted to demonstrate the real-time parameters for visible color changes, additional studies are warranted to further investigate this topic.

Nevertheless, *in vitro* studies may provide an initial estimate. This estimate was calculated on the basis of a research^26^ in which, according to coffee producers, the average time spent to consume a cup of coffee is 15 minutes and the average number of cups of coffee consumed per day is 3.2 cups. Therefore, 24 hours of immersion in coffee represents a monthly consumption of coffee. Thus, 21 days of immersion *in vitro*, as used in this study, simulates the susceptibility of ceramic brackets to coffee staining within 1 year and 9 months of orthodontic treatment.

This calculation can be individualized for each person, thereby allowing the risk of color change to be estimated according to orthodontic treatment time expected by the orthodontist and information on average color change provided by *in vitro* studies.

Akyalcin et al^19^ conducted an *in vitro* study to reproduce the exposure time necessary for a dye drink to act on brackets inside the mouth. The total time of the experiment was 26 weeks (equivalent to 6 months), with daily exposures of 10 minutes alternated by baths of water at 37ºC. The amount of bracket exposure to dye solution differed according to the frequency of use of each drink, as limited by the authors. A total time ranging from 13 to 60 hours of exposure was used for the entire study period.

In any *in vitro* study, the biggest challenge is to reproduce the real conditions of the oral cavity. Under the conditions of our study, we are not able to provide the real time of bracket staining. Nevertheless, bracket structures should be improved by manufacturers in order to increase color stability and standardize the production process of these accessories. To this end, additional research about the possible factors that might promote color change of esthetic brackets is necessary, since the mechanism of staining is not clear in the literature.

## CONCLUSION

The methodology of this study let us to conclude that ceramic brackets undergo color change when exposed to solutions of coffee, black tea and red wine, drinks commonly present in people's diet. However, the same crystalline structure, either monocrystalline or polycrystalline, do not follow the same or a similar pattern in color change, but vary from manufacturer to manufacturer; thereby showing lack of standardization in the manufacturing process of these brackets. Coffee dye produced the most marked color changes after 21 days of immersion for most ceramic brackets assessed.
